# Identity Statuses and Psychosocial Adjustment in Chinese Adolescents: A Latent Profile Analysis

**DOI:** 10.3390/bs16081277

**Published:** 2026-07-27

**Authors:** Shuang Gao, Yiman Huang, Xifeng Xia, Xu Zhu, Xiaochun Xie, Wim Van den Noortgate

**Affiliations:** 1 Department of Applied Psychology, Guangzhou Medical University, Guangzhou 511436, China; gaoshuang@gzhmu.edu.cn (S.G.); huangyiman@stu.gzhmu.edu.cn (Y.H.); xu0326202205@163.com (X.Z.); 2 Department of Psychology, University of Chinese Academy of Sciences, Beijing 100049, China; 3 Institute of Psychology, Chinese Academy of Sciences, Beijing 100101, China; 4 School of Psychology, Northeast Normal University, Changchun 130024, China; 5 Faculty of Psychology and Educational Sciences, Catholic University of Leuven, 8500 Leuven, Belgium; wim.vandennoortgate@kuleuven.be; 6 Imec-Itec, Catholic University of Leuven, 3001 Leuven, Belgium

**Keywords:** identity statuses, psychosocial adjustment, adolescents, latent profile analysis

## Abstract

Adolescence is a critical period for identity formation, a core developmental task that significantly influences psychosocial adjustment. Although person-centered studies based on Crocetti’s three-factor model have identified distinct identity statuses across several cultural contexts, comparable evidence from Chinese adolescents remains limited. The current study aims to explore whether the three-factor model of identity could be found in a Chinese adolescent sample and to examine the relationship between identity statuses and psychosocial adjustment. A sample of 4666 adolescents ($M_{age}$ = 18.37; 49.9% females) completed the Utrecht-Management of Identity Commitments Scale. Latent profile analysis revealed five distinct identity statuses: Searching Moratorium, Foreclosure, Avoidant, Undifferentiated, and Diffusion. These statuses differed significantly in levels of anxiety, subjective well-being, and resilience. Overall, identity statuses were significantly associated with psychosocial adjustment. Notably, adolescents in the Foreclosure status exhibited the most favorable psychosocial outcomes; however, this pattern may reflect an adaptive response to the Chinese educational context rather than indicating a more mature stage of identity development.

## 1. Introduction

Self-identity refers to individuals’ perception of their authentic self, encompassing a subjective sense of self-consistency and continuity across various contexts and time. Self-identity plays a pivotal role in facilitating adolescents’ transition, including psychological, social, and biological transformations (Branje et al., 2021). In this period, adolescents face various developmental challenges, such as prompting individuals to reevaluate who they are, the factors shaping their identity, and their aspirations for the future (Kroger et al., 2010). Only by obtaining satisfactory answers can adolescents integrate a clear sense of identity and navigate the transition into adult roles and responsibilities with considerable ease (Erikson, 1968; Luyckx et al., 2008). In order to provide supportive interaction for fostering their identity development, it is important to draw upon contemporary models of identity development and explore the relationship between identity statuses and psychosocial adjustment (Park et al., 2024).

### 1.1. Identity Processes, Statuses, and Profiles

Crocetti et al. (2008b) proposed a revised process model of identity formation, which serves as the guiding framework for this study. Their model incorporates three factors: commitment, in-depth exploration, and reconsideration of commitment (Crocetti et al., 2008a; Meeus et al., 2010). Commitment involves making firm choices and actively engaging in an identity alternative; in-depth exploration refers to examining one’s already chosen identity; and reconsideration of commitment involves contemplating alternative commitments to the current choice (Crocetti et al., 2015). This tripartite model expands Marcia’s (1966) identity status paradigm. Rooted in Erikson’s (1950) theoretical proposition of ego identity, Marcia (1966) advanced the identity status model by delineating two fundamental dimensions: exploration, defined as the search for meaningful alternatives in goals and values, and commitment, defined as the investment in specific chosen alternatives. Although this two-dimensional model laid the groundwork for identity status research, it is limited by its static, categorical nature and its lack of process orientation (Crocetti, 2017). The tripartite model thus enables a more nuanced examination of identity as a dynamic configuration rather than a static outcome, supporting the use of latent profile analysis to identify distinct identity statuses among adolescents.

Combining these three identity processes allows for the empirical extraction of five identity statuses. Specifically, Achievement: Individuals in this status have high commitment and in-depth exploration, low reconsideration of commitment. Foreclosure (also referred to as Closure in some presentations of the model; Crocetti, 2018): Individuals in this status have moderate commitment, low in-depth exploration, and low reconsideration of commitment. Moratorium: Individuals in this status cease commitments and explore until finding a considerably satisfactory choice, combined with low commitment, a medium level of in-depth exploration and a high reconsideration of commitments. Searching Moratorium: The status involves individuals who would maintain commitments and explore existing decisions although they doubt it, and have high levels of process of commitments, high in-depth exploration, and high reconsideration of commitment. Diffusion: These individuals have low commitment, low in-depth exploration, and low reconsideration of commitment (Crocetti, 2018). It is important to distinguish the Diffusion status identified in the present study through latent profile analysis from the construct of pathological identity Diffusion elaborated within the psychodynamic tradition. O. F. Kernberg (1984) conceptualized identity Diffusion as a core structural feature of borderline personality organization. P. F. Kernberg et al. (2000) further articulated criteria for distinguishing normative from pathological identity Diffusion during adolescence. The Psychodynamic Diagnostic Manual (PDM-2; Lingiardi & McWilliams, 2017; PDM-3, Lingiardi & McWilliams, 2025) integrates the assessment of identity formation within a multiaxial framework of personality functioning, thereby differentiating normative adolescent identity fluidity from structural disturbances. In contrast, the Diffusion status in this study is defined behaviourally and dimensionally, derived from self-report scores on identity process measures, and does not carry the clinical implications of pathological identity Diffusion in the Kernberg or PDM sense.

Previous research has applied a person-centered approach—which treats the individual as the primary unit of interest and assumes that relatively homogeneous profiles emerge in a coherent manner (Meeus et al., 2010; Rowley et al., 2003)—to identify identity statuses based on Crocetti’s three-factor model. Such studies have consistently identified five or six identity statuses across adolescent samples from the Netherlands (Crocetti et al., 2008a), Belgium (Verschueren et al., 2017), Hungary (Rivnyák et al., 2022), Japan (Hatano et al., 2016), and Turkey (Morsunbul et al., 2016). In some of these samples, additional profile types that do not correspond directly to the five theoretically specified statuses have been identified (Rivnyák et al., 2022; Verschueren et al., 2017). The most common are Undifferentiated, characterized by scores near the mean across all three identity processes, and Avoidant, characterized by relatively low commitment and in-depth exploration together with moderate reconsideration. However, comparable research among Chinese adolescents remains limited. Chinese senior high school students face a highly structured, examination-centered educational environment in which academic performance is closely tied to future opportunities (Zhao & Chang, 2024), conditions that may shape commitment and exploration processes in ways not yet examined. Although the U-MICS has demonstrated measurement invariance in Chinese samples (Crocetti et al., 2015), to our knowledge, no prior study has applied a person-centered approach to U-MICS data among Chinese adolescents.

### 1.2. Identity Formation Is Associated with Psychosocial Adjustment

Early conceptualizations of adjustment emphasized individuals’ ability to independently manage daily life and fulfill social responsibilities in alignment with age-related, social, and cultural expectations (Bergman, 2002). Contemporary identity research has expanded this concept into psychosocial adjustment, integrating internal psychological functioning with observable social adaptation (Crocetti et al., 2008a; Meeus et al., 2012). In the present study, psychosocial adjustment is defined as the degree to which individuals function adaptively in their emotional and psychological lives, encompassing both the presence of positive functioning and the relative absence of maladaptive distress (Branje et al., 2021). Consistent with this conceptualization, identity development has been linked to a broad range of psychosocial adjustment outcomes, including indicators of maladjustment (e.g., anxiety, depressive symptoms) and indicators of positive adjustment (e.g., subjective well-being, self-esteem; Branje et al., 2021). Meeus et al. (2012) examined the identity status model proposed by Waterman (1982) in 5-wave study of adolescents, suggesting that identity is not a stable disposition but develops from Diffusion toward achievement. Research has consistently indicated that adolescents with stable and robust identity commitments tend to report higher levels of psychosocial adjustment over time compared with those grappling with ongoing identity uncertainty (Crocetti et al., 2008a; Hatano et al., 2018; Meeus et al., 2010). That is, a more developed identity allows an individual to exhibit greater psychological flexibility in their functioning. Accordingly, the present study assessed psychosocial adjustment through three complementary indicators, capturing both the negative and positive facets of the construct as well as individuals’ adaptive resources. Anxiety, an emotional state experienced when coping with stress, reflects negative emotional adjustment (Zung, 1971). Subjective well-being, reflecting individuals’ overall satisfaction with life and their experience of positive emotion, represents positive subjective functioning (Campbell, 1976). Resilience, referring to the ability to restore a positive mental state when facing adversity, reflects the capacity to maintain or restore adaptive functioning under stress (Hu & Gan, 2008). Together, these three indicators provide a more comprehensive representation of psychosocial adjustment than any single indicator alone.

Some studies have explored the relationship between self-identity and these three indicators of psychosocial adjustment. Marcia’s (1966) early research on self-identity utilized anxiety to validate the existence of distinct identity statuses. Subsequent investigations have revealed that individuals in the Foreclosure status exhibited lower anxiety levels, whereas those in the Moratoriums and Diffusion statuses had higher anxiety levels (Lillevoll et al., 2013). Other studies have discovered relations between the three factors of Crocetti’s identity model and psychosocial adjustment: higher commitment displays a negative correlation with anxiety, whereas a higher level of in-depth exploration shows a positive correlation with anxiety (Crocetti et al., 2015). Furthermore, early research has indicated a close association between a more developed identity and elevated subjective well-being (Hatano et al., 2020). Commitment has positive effect on high life satisfaction, and the reconsideration of commitment has a negative effect on life satisfaction (Hirschi, 2012). Lastly, resilience can affect how adolescents derive meaning from their situation and identity (Koni et al., 2019; Thompson et al., 2019). Koni et al. (2019) suggested that developing social identity can foster resilience in adolescents, particularly those facing adversity.

### 1.3. Gender and Age Differences in Identity Statuses

Self-identity development during adolescence exhibits variations based on gender and age. Regarding gender, Erikson (1968) initially emphasized gender disparities in self-identity development, suggesting that physiological differences contribute to women’s earlier physical and psychological maturity compared to men (Colom & Lynn, 2004), thereby influencing identity development. Research has indicated that women tend to have a more developed identity than men (Hatano et al., 2018). However, subsequent research has revealed markedly nuanced gender differences, challenging the notion that women consistently demonstrate a superior identity development (Verschueren et al., 2017). In the context where men are generally expected to be independent and confident, while women are expected to manage social relationships well, social and cultural factors play an important role in gender differences. Concerning age, there is a general belief that identity development improves gradually over time, but instances exist when some groups stagnate at a particular identity status (Morsunbul et al., 2016).

### 1.4. Chinese Youth

Culture shapes the self, which in turn plays a critical role in the formation and development of culture. Self-construal, individualistic cultures emphasize independence, uniqueness, and free choice, while collectivist cultures stress interdependence, social embeddedness, and obligations and loyalty to in-groups, such as family (Huang et al., 2020; Grossmann & Na, 2014). Traditionally, China is viewed as a collectivist culture that values and reinforces traits such as modesty, respect for authority, and concern for relational harmony among youth (Cai et al., 2012). Research has found that valuing family and friends, as well as emphasizing responsibility and altruism, is robust attitudes in China (Zeng & Greenfield, 2015). Moreover, Confucianism emphasizes educational achievement and hierarchical authority. Learning is regarded as the primary task of students, and the notion that academic performance and success are closely linked is instilled in students from an early age. In this context, commitment may be influenced by external pressure and may reflect compliance rather than a choice guided by one’s own inner desires. At the same time, the Chinese educational environment is less likely to encourage students to engage in activities beyond learning, and in-depth exploration and reconsideration of commitment may therefore face more resistance than commitment. Over the past several decades, with the global increase in individualism due to modernization (Greenfield, 2013), China’s diverse modes of production and ecological environments result in a cultural pattern that includes both individualistic and collectivistic orientations (Leung, 2008). Additionally, in the context of China’s exam-oriented education system, adolescents are required to focus on their studies, which reduces their opportunities to explore personal interests and pursuits. Consequently, understanding the process of identity formation in Chinese youth becomes necessary due to the clash between traditional values and the rise of individualism.

There are relatively few studies on identity development among Chinese adolescents, and the consequences of the tension between traditional collectivist values and rising individualism for identity formation remain largely unexplored. Fan and Li (2025) examined the developmental trajectories of identity coherence and identity confusion in Chinese adolescents from sixth to ninth grade, and found that the two did not follow parallel courses. Identity coherence decreased across this period, as adolescents began to move away from passively accepting parental values toward actively exploring and forming their own; identity confusion, by contrast, increased through eighth grade before gradually declining as academic goals became clearer. Wu et al. (2023) conducted a qualitative study of identity among Chinese adolescents with mental disorders and found that traditional Confucian emphases on educational achievement and hierarchical authority permeated family upbringing. This was reflected in parents’ use of harsh criticism to pressure their children academically—an authoritarian parenting style that, when combined with adolescents’ limited coping capacities, may further undermine self-esteem and hinder the development of a coherent sense of identity. Together, these studies highlight the relevance of academic environments and cultural factors to identity development among Chinese youth. However, existing research has predominantly adopted variable-centered approaches and has rarely used person-centered methods such as latent profile analysis to depict identity profiles in this population.

### 1.5. Present Research

The present study had three aims. The first aim was to examine whether the five identity statuses identified in prior person-centered studies (Crocetti et al., 2008a, 2008b; Meeus et al., 2010) could also be identified among Chinese adolescents; given the person-centered nature of this question, no a priori hypothesis was specified regarding the number or composition of profiles. The second aim was to examine gender and age differences across identity statuses. Based on prior findings (Hatano et al., 2018; Morsunbul et al., 2016), we expected females to be more represented in statuses characterized by stronger commitment; given the restricted age range of the sample, age differences were examined on an exploratory basis. The third aim was to examine how identity statuses relate to psychosocial adjustment. Consistent with previous research linking firm commitments to better adjustment (Meeus et al., 2010), we expected statuses characterized by higher commitment to show lower anxiety and higher subjective well-being and resilience, and statuses characterized by low commitment to show the opposite pattern.

## 2. Methods

### 2.1. Participants and Procedure

A cross-sectional survey design was used to examine identity profiles and their associations with psychosocial adjustment among Chinese Grade 12 students. Participants were recruited from 79 senior high schools located in coastal cities of Zhejiang Province, China. The participants were in late adolescence, a period that recent developmental perspectives consider to extend into the early twenties (Sawyer et al., 2018) and that is characterized by significant identity exploration and consolidation. Zhejiang Province was selected for data collection because of the accessibility of school-based recruitment and the relatively standardized educational context experienced by senior high school students in China, particularly during preparation for the national college entrance examination. Participants were recruited using a school-based convenience sampling approach. The inclusion criteria were as follows: (a) enrollment as a Grade 12 student at one of the participating high schools; (b) provision of written informed consent prior to participation; and (c) completion of the self-report questionnaires independently. The exclusion criterion was substantial missing questionnaire data that prevented reliable scoring of one or more study measures. Data were screened for completeness prior to analysis. Of the 4723 students initially surveyed, 57 (1.2%) were excluded because they failed to complete one or more of the study’s core measures in their entirety (i.e., an entire scale was left unanswered), precluding reliable scoring of those measures. The final sample consisted of 4666 students. From this sample, 2336 (50.1%) were males and 2330 (49.9%) were females. Their ages ranged from 16 to 23 years (mean = 18.37 years; *SD* = 0.54 years).

Although a subset of participants were aged 19 years or older (*n* = 1564 for age 19 years; *n* = 65 for age ≥ 20 years), all participants were in their final year of senior high school and experienced the same educational context. In terms of the parents’ education level, 68.8% of the fathers and 65.9% of the mothers had a bachelor degree or higher. In terms of the family structure, 70.5% of the participants were from intact families, and 76.6% of participants were from only-child families.

Participation was voluntary and anonymous. Participants were informed about the purpose of the study and asked for their informed consent. The anonymity of the study was emphasized before data collection. Data were collected during a regular class period. Participants completed the questionnaires in their classrooms or computer rooms under the supervision of trained researchers. No teaching staff members were present. Research procedures were approved by the Research Ethics Committee of Taizhou Academy of Educational Monitoring and Sciences (Protocol Number: 202103). The adolescents and their parents were informed about the aims and procedures of the study and were invited to participate voluntarily. Written informed consent was obtained from parents or legal guardians for participants under 18 years of age. In addition, assent was obtained from all adolescent participants prior to data collection. For participants aged 18 years or older, informed consent was obtained directly from the participants themselves. Participation was entirely voluntary, and no monetary compensation, gifts, or other incentives were provided.

### 2.2. Measures

#### 2.2.1. Identity

The three factors of Crocetti’s model were measured by means of the Utrecht-Management of Identity Commitments Scale (U-MICS; Crocetti et al., 2008b). This scale consists of 26 items including 10 items of the commitment subscale, 10 items of the in-depth exploration subscale and 6 items of the reconsideration of commitment subscale. Each scale has ideological (i.e., education) and interpersonal (i.e., friendships) domains. All items are rated on a 5-point Likert scale ranging from 1 (completely not true) to 5 (completely true). Sample items are: “My education gives me certainty in life” (ideological commitment), “I often think it would be better to try to find a different best friend” (interpersonal reconsideration of commitment). The U-MICS has been translated, validated, and used in many languages (Crocetti et al., 2015), Cronbach’s alphas were 0.85 for commitment, 0.70 for in-depth exploration, and 0.72 for reconsideration of commitment in Chinese version. In this study, Cronbach’s alphas were 0.97 for commitment, 0.89 for in-depth exploration, and 0.88 for reconsideration of commitment. The internal consistency for commitment (0.97) was notably higher than that previously reported for the Chinese version of the U-MICS (0.85; Crocetti et al., 2015), which may partly reflect item homogeneity and the large, relatively homogeneous sample. The factorial structure of the U-MICS was examined using confirmatory factor analysis (CFA). Consistent with the original design of the scale, which distinguishes an ideological and an interpersonal domain within each identity process (Crocetti et al., 2008b), a six-factor model was tested (i.e., ideological and interpersonal commitment, in-depth exploration, and reconsideration of commitment). CFA was conducted using maximum likelihood estimation, and the five-point Likert-type items were treated as continuous indicators. Model fit was evaluated using the comparative fit index (CFI), the Tucker–Lewis index (TLI), and the root mean square error of approximation (RMSEA). The results were: *χ*^2^(284) = 13,679.835, *p* < 0.001, CFI = 0.916, TLI = 0.904, RMSEA = 0.101. The comparative fit indices approached conventional thresholds, whereas the RMSEA exceeded the conventional 0.08 criterion, indicating residual model misfit that should be considered when interpreting the findings. Considering the satisfactory standardized factor loadings (0.631–0.981) and previous evidence supporting the structure and measurement invariance of the U-MICS in Chinese samples (Crocetti et al., 2015), the six-factor model provided preliminary support for the intended measurement structure in the present sample; however, the findings should be interpreted cautiously given the non-optimal model fit. Because the present study aimed to identify general identity status profiles rather than domain-specific configurations, items from the two domains were aggregated within each identity process to obtain three overall indicators for the latent profile analysis, an approach adopted in previous research examining identity processes across domains (Meeus et al., 2012; Crocetti, 2018).

#### 2.2.2. Anxiety

Anxiety was assessed with the Self-Rating Anxiety Scale (SAS; Zung, 1971), which has been widely used in clinical practice and has demonstrated good reliability and validity. The SAS consists of 20 items, each rated on a 4-point Likert scale ranging from 1 (a little of the time) to 4 (most of the time); item scores are summed to obtain a total raw score. Following the original scoring procedure, the raw score multiplied by 1.25 yields a standard score used solely for clinical interpretation of symptom severity, with standard scores of 53–62, 63–72, and above 72 indicating mild, moderate, and severe anxiety, respectively. In the present study, raw scores were used for all statistical analyses involving anxiety; the clinical cut-offs were not used as analytical variables. The Chinese version of the SAS has been shown to be valid and reliable, with a Cronbach’s alpha of 0.76 (Xu et al., 2011). In the current study, Cronbach’s alpha was 0.89.

#### 2.2.3. Subjective Well-Being

Subjective well-being was assessed by the Index of Well-Being (IWB; Campbell, 1976), which includes 9 items. All items are rated on a 7-point Likert scale ranging from 1 (bored) to 7 (interested). Item scores of IWB were simply added up, generating a total support score of 9 to 63, with higher scores indicating higher index of well-being (question is “How do you feel about life in the past few weeks?”). This scale has been adapted to Chinese culture and widely used among Chinese youth (Xue & Li, 2026). The internal consistency was 0.96. Cronbach’s alpha of the scale in the current study was 0.97. This similarly high value may reflect item homogeneity and the characteristics of the present sample.

#### 2.2.4. Resilience

Resilience was assessed by the Resilience Scale for Chinese Adolescents (RSCA; Hu & Gan, 2008). This scale consists of 27 items measuring two dimensions: Personal strength (15 items), and Support strength (12 items). All items were rated on a 5-point Likert scale ranging from 1 (never) to 5 (always). Item scores of RSCA were simply added up, generating a total support score of 27 to 135, with higher scores indicating higher resilience (questions such as “Failure always makes me lose confidence and courage.”). The scale has good internal consistency, and Cronbach’s alpha was 0.83 in Chinese samples (Hu & Gan, 2008). Cronbach’s alphas was 0.87 of the whole scale in the current study.

#### 2.2.5. Data Analysis

After exploring (relationships between) the variables using correlations and other descriptive statistics, the scores obtained on the three subscales of the U-MICS were submitted to a Latent Profile Analysis (LPA), allowing us to identify homogenous subgroups of individuals with regard to identity. LPA is a person-centered approach used to identify distinct latent subgroups within a sample (Oberski, 2016). Because LPA accounts for measurement error, it is superior to cluster analysis. Unlike Latent Class Analysis, LPA uses continuous indicators (such as items from the three identity factors) instead of categorical ones (Wen et al., 2023). LPA can be effectively applied to classify identity statuses among Chinese adolescents.

The following statistical indices were used to choose the best fitting model with regard to the number of profiles within the sample: the log likelihood (LL), the Bayesian information criterion (BIC), the sample-size adjusted Bayesian information criterion (SSA-BIC), the Akaike information criterion (AIC), the Lo–Mendell–Rubin likelihood ratio test (LMR-LRT), and the bootstrap likelihood ratio test (BLRT). Furthermore, the entropy was used to assess the overall quality of the final classification. The smaller the AIC, BIC and SSA-BIC, and the closer the Entropy to 1, the better the fit. In addition, LMR and BLRT can be used to compare the difference in fit of two nested models, i.e., a model with *k* profiles and a model with *k*-1 profiles: if the above two values reach a significant level, it indicates that the model with the current number of profiles (*k*) is significantly better than the model with *k*-1 profiles (Muthén & Muthén, 2017). Although some prior studies have recommended that each profile contain at least 5% of the sample or a minimum of 30 participants (Li et al., 2017), current recommendations in latent profile analysis treat class size as one consideration to be weighed together with statistical fit, classification quality, parsimony, and theoretical interpretability, rather than as an absolute exclusion rule (Nylund et al., 2007; Spurk et al., 2020).

After the best fitting model was chosen, as an auxiliary validation procedure, discriminant analysis was conducted to examine the separability of the identified profiles. In the discriminant analysis, the clusters resulting from the LPA were the dependent variable, and the independent variables were the scores of the three subscales of the U-MICS. After learning the relation between the group variable and the independent variables, it reclassified each participant in a cluster. Next, the prediction accuracy of each cluster was calculated, defined as the proportion of participants categorized by the LPA in a cluster that were categorized in the same cluster by the discriminant analysis. The clusters are named based on their average score on the three U-MICS subscale scores.

Next, differences between clusters in sociodemographic variables were studied further. For the categorical variable gender, the profiles were contrasted by conducting $\chi^{2}$ tests. For the quantitative variable age, an analysis of variance (ANOVA) was done.

Finally, differences in anxiety, subjective well-being, and resilience-related measures across identity status groups were examined. A two-way multivariate analysis of variance (MANOVA) was conducted with anxiety, subjective well-being, personal strength, and support strength as dependent variables. Identity status (Diffusion, Undifferentiated, Avoidant, Foreclosure, and Searching Moratorium), gender, and their interaction were entered as fixed factors. The total resilience score was not included in the MANOVA because it represents a composite score derived from the two resilience dimensions and may introduce redundancy among dependent variables when analyzed together with its component subscales (Tabachnick & Fidell, 2019). Instead, total resilience was analyzed separately using univariate analysis. Significant multivariate effects were followed by follow-up univariate analyses to identify specific dependent variables contributing to the observed effects. Given the large sample size (and hence the large statistical power), we used the more conservative value of 0.01 as level of significance in order to reduce probability of Type I error (i.e., rejecting the null hypothesis when the null hypothesis is true).

Statistical analyses were conducted using IBM SPSS Statistics version 27.0 (IBM Corp., Armonk, NY, USA), except for LPA, which was performed using Mplus version 8.0 (Muthén & Muthén, Los Angeles, CA, USA).

## 3. Results

### 3.1. Correlation Analyses

Descriptive statistics for the study variables and the relations between them are presented in Table 1. Except for the fact that there was no significant correlation between reconsideration of commitment and resilience, all variables were significantly correlated.

### 3.2. Latent Profile Analyses

To identify the optimal number of self-identity profiles among high school students, latent profile models with two to seven profiles were estimated using the three subscale scores of the identity scale (commitment, in-depth exploration, and reconsideration of commitment) as indicators. The fit indices for all estimated models are presented in Table 2. Model selection was based on multiple criteria, including information criteria (AIC, BIC, and SSA-BIC), likelihood ratio tests (LMR-LRT and BLRT), entropy, profile size, parsimony, and theoretical interpretability, as recommended in previous latent profile analysis studies (Nylund et al., 2007; Spurk et al., 2020). The results showed that AIC, BIC, and SSA-BIC values continuously decreased as the number of profiles increased, indicating improved statistical fit with the addition of profiles. However, information criteria were not considered as the sole basis for model selection because more complex models generally tend to demonstrate improved fit. The BLRT was significant across all tested solutions, whereas the LMR-LRT was significant only for the two-profile and five-profile solutions, suggesting that the five-profile solution provided a statistically supported improvement over the four-profile solution. Although the three- and four-profile solutions demonstrated acceptable statistical fit and interpretable patterns, they provided a relatively broad classification of identity development patterns and did not capture several theoretically meaningful subgroups identified in the five-profile solution. Conversely, although the six- and seven-profile solutions showed lower AIC, BIC, and SSA-BIC values, the additional profiles primarily represented subdivisions of existing profiles rather than qualitatively distinct identity patterns, while also introducing profiles with smaller sample sizes and limited theoretical distinctiveness. The five-profile solution demonstrated good classification quality (entropy = 0.970) and yielded theoretically meaningful identity patterns consistent with Marcia’s identity status theory. Although two profiles contained relatively small proportions of participants (2.2% and 3.7%), these profiles represented theoretically meaningful identity statuses and provided important information regarding less prevalent identity configurations. Therefore, profile size was considered as one criterion rather than an absolute exclusion rule, as there is no universally accepted minimum class size criterion for latent profile analysis (Nylund et al., 2007; Spurk et al., 2020). Taken together, considering statistical fit indices, likelihood ratio tests, classification quality, profile size, parsimony, and theoretical interpretability, the five-profile solution was selected as the final model.

To further examine the distinctiveness and classification consistency of the identified latent profiles, discriminant analysis was conducted using the three identity indicators included in the LPA. As Table 3 shows, the results showed that in the first cluster of identity, 91 participants from the 102 participants in the first LPA cluster (this is 89.2%) were categorized in the same cluster by the discriminant analysis; in the second, third, fourth and fifth cluster, an accuracy rate of 90.4%, 93.5%, 100% and 100% was found. Overall, a total of 4367 participants were categorized in the same cluster as LPA by the discriminant analysis, with a prediction accuracy of 93.6%. These findings suggest that the identified profiles showed clear differentiation based on the identity indicators used in the LPA.

### 3.3. Describing the Identity Clusters

**Figure 1:**
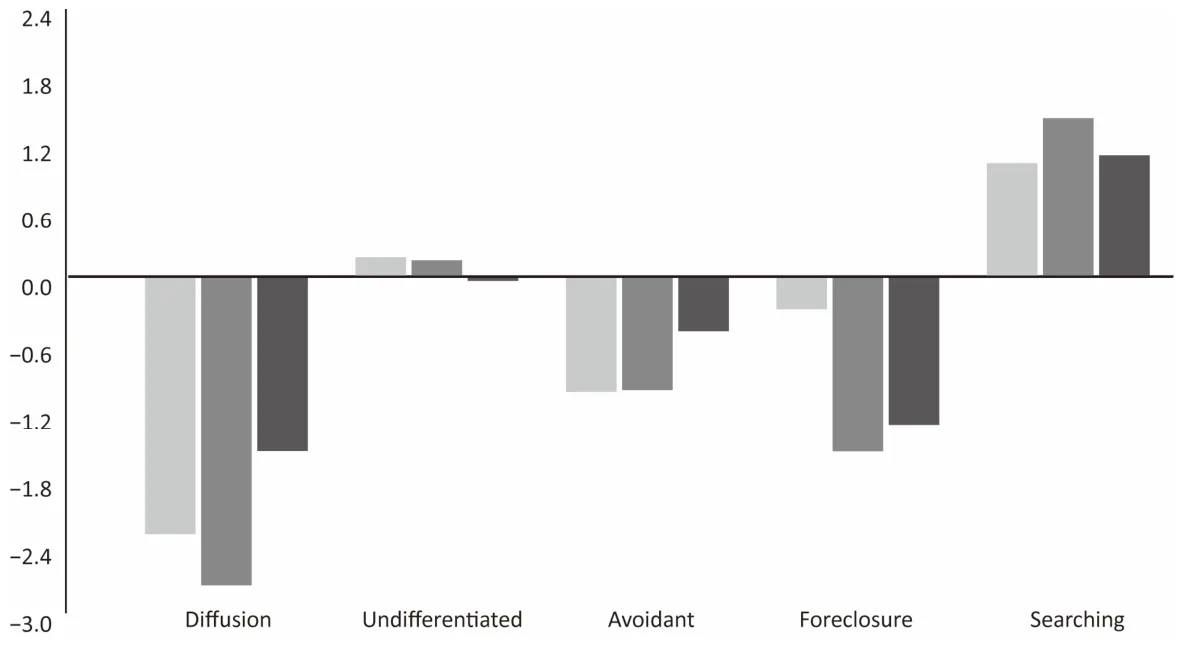
Z-scores for commitment, in-depth exploration and reconsideration of commitment for the five identity statuses.

The standardized scores for commitment, in-depth exploration, and reconsideration of commitment for the five clusters are shown in Figure 1. Drawing upon past research (Morsunbul et al., 2016; Crocetti et al., 2008a, 2008b; Meeus et al., 2010), we assigned cluster labels based on the standardized scores for the three identity factors within each cluster. We named cluster 1 the Diffusion identity status (*n* = 102; 2.2%), which was characterized by very low scores on commitment, in-depth exploration and reconsideration of commitment factors. We named cluster 2 the Undifferentiated identity status (*n* = 2161; 46.3%), which was characterized by moderate scores on commitment, in-depth exploration and reconsideration of commitment factors. We named cluster 3 the Avoidant identity status (*n* = 1252; 26.8%), which was characterized by moderate low scores on commitment and in-depth exploration factors, as well as score near the mean on reconsideration of commitment factor. We named cluster 4 the Foreclosure identity status (*n* = 174; 3.7%), which was characterized by moderate low scores on in-depth exploration and reconsideration of commitment factors, as well as score near the mean on commitment factor. We named cluster 5 the Searching Moratorium identity status (*n* = 977; 20.9%), which was characterized by moderate high scores on commitment, in-depth exploration and reconsideration of commitment factors. These labels are consistent with those used in prior person-centered studies that have identified comparable profiles from U-MICS data. Specifically, the Undifferentiated and Avoidant profiles have been reported in LPA studies of Turkish (Morsunbul et al., 2016) and Belgian (Verschueren et al., 2017) adolescent samples, and both labels are descriptive terms for empirically derived clusters whose score configurations do not correspond directly to the five theoretically specified statuses in Crocetti’s model. The Avoidant label in this context refers to a pattern of low engagement in identity processes (moderate low commitment and in-depth exploration, with moderate reconsideration) and should not be conflated with Berzonsky’s Diffuse-Avoidant identity style, which derives from a different theoretical and measurement tradition.

### 3.4. Gender and Age Differences on Identity Statuses

We performed Chi-square tests to examine whether participants’ distribution in the five identity statuses differed according to gender. Results indicated differences by gender in the distribution of participants across the five identity clusters *χ*^2^ (*df* = 4, *n* = 4666) = 112.876, *p* < 0.001, Cramer’s *V* = 0.156. As reported in Table 4, females were more likely to be classified in the Foreclosure and Undifferentiated statuses than males. Furthermore, males were more represented than females in the Diffusion and Searching Moratorium statuses. With respect to age, we used a one-way ANOVA to analyse the age differences among five identity statuses. The results showed that *F* (4, 4661) = 0.491, *p* = 0.743, which means there were no significant differences in age among identity statuses.

### 3.5. Identity Statuses, Anxiety, Subjective Well-Being and Resilience

We conducted a two-way MANOVA with anxiety, subjective well-being, personal strength, and support strength as dependent variables. Identity status (Diffusion, Undifferentiated, Avoidant, Foreclosure, and Searching Moratorium) and gender were entered as fixed factors, and the identity status × gender interaction was also examined. The total resilience score was analyzed separately using univariate analysis because it represents a composite score derived from the two resilience dimensions and was therefore not included in the multivariate model to avoid redundancy and excessive overlap among dependent variables arising from including both a composite score and its component subscales in the same multivariate model. At the multivariate level, identity status showed a significant main effect on the combined dependent variables, Wilks’ λ = 0.751, *F* (16, 14,215.787) = 87.334, *p* < 0.001, η_p_^2^ = 0.069. However, the identity status × gender interaction was not significant, Wilks’ λ = 0.995, *F* (16, 14,215.787) = 1.400, *p* = 0.131, η_p_^2^ = 0.001.

### 3.6. Anxiety

Follow-up univariate analyses of the variables included in the MANOVA revealed significant main effects of the identity statuses on anxiety, *F* (4, 4656) = 36.846, *p* < 0.001, η_p_^2^ = 0.031. Tukey post hoc comparisons (see Table 4) showed that individuals in the Diffusion and Avoidant identity statuses scored highest on anxiety, followed by Undifferentiated peers. And the individuals in Foreclosure and Searching Moratorium identity statuses scored the lowest on anxiety. At the univariate level, the identity status × gender interaction effect on anxiety did not reach the prespecified significance threshold (*p* < 0.01), *F* (4, 4656) = 3.111, *p* = 0.014, η_p_^2^ = 0.003.

### 3.7. Subjective Well-Being

Follow-up univariate analyses of the variables included in the MANOVA revealed significant main effects of the identity statuses on subjective well-being, *F* (4, 4656) = 225.021, *p* < 0.001, η_p_^2^ = 0.162. Tukey post hoc comparisons (see Table 4) showed that individuals in the Foreclosure and Searching Moratorium identity statuses scored higher on subjective well-being, followed by Undifferentiated identity status peers. And the individuals in Diffusion and Avoidant identity statuses scored lower on subjective well-being. At the univariate level, the identity status × gender interaction effect on subjective well-being was statistically not significant, *F* (4, 4656) = 1.253, *p* = 0.286, η_p_^2^ = 0.001.

### 3.8. Resilience

Follow-up univariate analyses of the variables included in the MANOVA revealed significant main effects of identity status on personal strength, *F* (4, 4656) = 323.753, *p* < 0.001, η_p_^2^ = 0.218, and support strength, *F* (4, 4656) = 166.801, *p* < 0.001, η_p_^2^ = 0.221. Tukey post hoc comparisons (see Table 4) showed that individuals in the Foreclosure identity status group scored highest on personal strength, followed by those in the Searching Moratorium identity status group. Individuals in the Undifferentiated identity status group scored lower than those in the Searching Moratorium group, whereas individuals in the Diffusion and Avoidant identity status groups scored the lowest. For support strength, individuals in the Foreclosure and Searching Moratorium identity status groups scored highest, followed by those in the Undifferentiated identity status group, whereas individuals in the Diffusion and Avoidant identity status groups scored the lowest. At the univariate level, the identity status × gender interaction was not significant for either personal strength, *F* (4, 4656) = 0.863, *p* = 0.490, η_p_^2^ = 0.001, or support strength, *F* (4, 4656) = 0.982, *p* > 0.050, η_p_^2^ = 0.001.

In addition, because the total resilience score was not included in the MANOVA, it was analyzed separately using a univariate analysis. A significant main effect of identity status was observed for total resilience, *F* (4, 4656) = 329.915, *p* < 0.001, η_p_^2^ = 0.221. Tukey post hoc comparisons (see Table 4) showed that individuals in the Foreclosure identity status group scored highest on total resilience, followed by those in the Searching Moratorium identity status group. Individuals in the Undifferentiated identity status group scored lower than those in the Searching Moratorium group, whereas individuals in the Diffusion and Avoidant identity status groups scored the lowest. The identity status × gender interaction effect on total resilience was not significant, *F* (4, 4656) = 1.077, *p* = 0.366, η_p_^2^ = 0.001.

## 4. Discussion

This study’s initial objective was to determine whether or not the five identity statuses could be identified within a sample of Chinese adolescents. Results revealed the presence of the five statuses of identity (i.e., Diffusion, Undifferentiated, Avoidant, Foreclosure, and Searching Moratorium) among the Chinese youth. Note that the largest proportion of individuals fell within the Undifferentiated status, comprising half of the sample. Individuals in the Avoidant and Searching Moratorium statuses followed, accounting for 26.8% and 20.9%, respectively, while under 10% of the population were categorized into the Foreclosure and Diffusion statuses.

Prior research has also used the LPA method to delineate adolescent identity statuses into five or six categories (Crocetti et al., 2008a; Verschueren et al., 2017; Rivnyák et al., 2022; Hatano et al., 2016), albeit with variations in specific identity types. The Achievement status did not emerge in our study. One possible explanation relates to features of the Chinese educational system, in which academic diligence is often treated by teachers and parents as students’ primary task, and examination performance is closely tied to notions of success (Zhao & Chang, 2024). It is also possible that a growing emphasis on individual development, together with wider exposure to alternative paths to career success, has introduced some tension into how students relate to the traditional student role during this final year of secondary school. This tension may be particularly difficult to resolve within an educational environment characterized by mandatory learning demands and limited guidance in personal self-exploration, conditions that may constrain students’ ability to integrate the identity-related conflicts characteristic of the Searching Moratorium phase. We speculate that this tension may help explain why so few students in our sample resolved commitment and exploration into the high-commitment, high-exploration configuration that defines Achievement, and instead remained distributed across the other four statuses at varying levels of internal conflict and exploration. This interpretation is necessarily speculative, as the present study did not directly assess these educational or sociocultural conditions. Notably, in the Chinese cultural context, commitment may be characterized more by compliance than by genuine internalization, yet the instruments used in this study cannot distinguish between these two forms (Ouyang et al., 2021).

The second objective of this study was to investigate potential differences in age and gender among the five identity statuses. Contrary to previous findings (Morsunbul et al., 2016; Verschueren et al., 2017), the results of this study suggest no significant age differences between statuses. This may be attributable to the concentration of our sample on senior three students, resulting in a narrow age distribution. In our findings, females outnumbered males in the Foreclosure and Undifferentiated statuses, whereas males outnumbered females in the Diffusion and Searching Moratorium statuses. Previous research has also noted a higher proportion of males in the Diffusion status at this age. This pattern may reflect the earlier mental and physical maturation of females relative to males, who tend to mature later in adolescence (Klimstra et al., 2010).

Notably, our findings also revealed a higher proportion of males in the Searching Moratorium status, diverging from earlier studies suggesting more female representation in this category (Meeus et al., 2012; Morsunbul et al., 2016). This result may reflect sociocultural expectations within the Chinese context, where females are often encouraged to conform to familial and institutional authority (Chen, 2017). Such normative expectations may foster stronger identity commitments in females, even in the absence of extensive self-exploration. While externally guided commitment can facilitate identity resolution, it may also constrain in-depth exploration and internal reflection. In contrast, males may experience greater latitude for autonomous exploration, potentially leading to increased identification with the Searching Moratorium status. The sociocultural mechanisms proposed here—such as differential familial expectations and gendered patterns of guided versus autonomous exploration—were not directly assessed in the present study. Accordingly, these interpretations should be viewed as possible explanations for the observed gender differences rather than conclusions supported by the current data.

Lastly, this study examined the correlation between identity statuses and psychosocial adjustment, as reflected in anxiety, subjective well-being, and resilience. Our findings indicated significant differences in anxiety, subjective well-being, and resilience across the identity statuses. Foreclosure showed the most favorable psychosocial adjustment in this sample, with the lowest anxiety levels, highest subjective well-being, and greatest resilience. Individuals in the Searching Moratorium status are characterized by low anxiety levels and higher subjective well-being, albeit with lower resilience than Foreclosure. Undifferentiated displayed moderate psychosocial adjustment among the five statuses, with anxiety, subjective well-being, and resilience scores falling in the mid-range. Conversely, the Diffusion and Avoidant statuses showed the poorest psychosocial adjustment, marked by the highest anxiety scores and lowest levels of subjective well-being and resilience. It is important to note that, unlike the pathological identity Diffusion described in O. F. Kernberg (1984) and the PDM framework (P. F. Kernberg et al., 2000; PDM-2; Lingiardi & McWilliams, 2017; PDM-3, Lingiardi & McWilliams, 2025), the Diffusion cluster identified in this study was defined behaviorally and dimensionally, based on self-reported identity process scores. Our data indicate that the Diffusion and Avoidant statuses were associated with higher anxiety at the behavioral level. However, because we did not assess clinical diagnostic indicators, we cannot determine whether any profile in this sample reflects clinically significant pathology.

In this study, the Foreclosure status showed the most favorable psychosocial adjustment. Prior research has indicated that high commitment is associated with lower anxiety, consistent with the lower anxiety observed in the Foreclosure status in our sample (Crocetti et al., 2008b; Meeus et al., 2010). However, the findings regarding subjective well-being and resilience appear to contrast with previous studies characterizing Foreclosure as relatively rigid and less developmentally mature (Kroger, 1993; Lillevoll et al., 2013). These divergent findings may reflect contextual adaptation within the Chinese senior high school environment, where students are strongly encouraged to prioritize mainstream academic values and maintain their educational commitments while engaging in relatively limited exploration of alternative identities. In such a context, positive external feedback—such as praise from parents and teachers and favorable academic evaluations—may enhance students’ subjective well-being and resilience (Kumpfer, 2002). It is important to note, however, that the present measures cannot distinguish commitments that are genuinely internalized from those maintained primarily through external compliance. Some individuals classified as Foreclosure may therefore appear well adjusted because they conform to external expectations rather than because they have achieved an integrated sense of identity. Accordingly, this favorable psychosocial adjustment should not necessarily be interpreted as indicating a more advanced stage of identity development.

The results for the Searching Moratorium, Undifferentiated, Diffusion, and Avoidant statuses were broadly consistent with prior findings linking identity development processes to psychosocial adjustment (Abu-Rayya, 2006; Waterman, 2007; Bogaerts et al., 2023). When individuals face uncertainty about the future, maintaining strong commitments while remaining open to exploration appears to be associated with better psychosocial adjustment than disengaging from both (Crocetti, 2018).

### Implications, Limitations and Future Directions

The present findings indicate that the Achievement status was absent among the Chinese senior high school students in our sample and that identity status distributions varied by gender. Based on these findings, schools could enhance identity-related education and identify students with less adaptive identity profiles (e.g., Diffusion, Avoidant) as potential targets for support.

This study has several limitations. First, although the present study contributes to understanding identity development among Chinese adolescents, the sample was recruited from Grade 12 students in a single province (Zhejiang Province). Therefore, caution should be exercised when generalizing these findings to adolescents from other regions, educational stages, or sociocultural contexts. In addition, because the present study was based on cross-sectional data and a restricted age range, causal inferences cannot be drawn. Future studies should employ geographically diverse longitudinal samples to examine these processes over time, though longitudinal designs clarify temporal ordering but do not eliminate confounding; stronger causal conclusions would require experimental or intervention research. Second, the cultural and educational factors discussed in interpreting the absence of the Achievement status (e.g., exam-oriented schooling and exposure to alternative career paths via the Internet) were not directly assessed, nor were cultural orientation measured. Furthermore, in the Chinese cultural context, commitment may encompass both compliance-based and internalized forms, yet the instruments used in this study cannot distinguish between these two aspects (Ouyang et al., 2021). Future research should incorporate culturally sensitive measures of cultural orientation, and commitment to further clarify the developmental mechanisms underlying identity formation. Third, although this study examined differences in psychosocial adjustment across identity statuses, future research could incorporate the PDM-3 diagnostic framework (Lingiardi & McWilliams, 2025) to examine whether low identity integration is associated with clinically significant pathology. Fourth, although the five-profile solution was selected on theoretical and interpretability grounds, the fit indices did not unambiguously favor this solution over adjacent models, and alternative profile solutions may also provide an adequate representation of the data. Moreover, although confirmatory factor analysis supported a domain-specific six-factor structure, the profile analyses aggregated ideological and interpersonal domains to represent general identity statuses. Future research should examine domain-specific identity profiles that may have been obscured by this approach. Finally, although the discriminant analysis demonstrated high classification consistency, it should be interpreted cautiously because the same identity indicators used to derive the latent profiles were also used in the discriminant analysis. Therefore, this analysis reflects the distinctiveness of the identified profiles rather than providing an independent validation of the latent profile solution.

## 5. Conclusions

Using a person-centered approach, this study explored Chinese adolescents’ profiles of identity status from three factors (commitment, in-depth exploration and reconsideration of commitment) and examined how these profiles of identity status correspond with adolescents’ demographic characteristics and psychosocial adjustment. The results identified five profiles of identity status (i.e., Diffusion, Undifferentiated, Avoidant, Foreclosure, and Searching Moratorium). The largest proportion of individuals fell within the Undifferentiated status, comprising nearly half of the sample. Individuals in the Avoidant and Searching Moratorium statuses followed, while the Foreclosure and Diffusion statuses accounted for a small proportion of samples. Females were more likely to be classified in the Foreclosure and Undifferentiated statuses, whereas males were more likely to fall into the Diffusion and Searching Moratorium statuses. Among the five statuses of Chinese adolescents, Foreclosure showed the most favorable psychosocial adjustment, with the lowest anxiety level, highest subjective well-being, and greatest resilience, followed by Searching Moratorium status characterized by a low anxiety level and high subjective well-being level, albeit with lower resilience than Foreclosure. Undifferentiated status reported moderate psychosocial adjustment, with anxiety, subjective well-being, and resilience scores falling in the mid-range. Diffusion and Avoidant statuses exhibited the poorest psychosocial adjustment, with the highest anxiety level and the lowest level of subjective well-being and resilience. Overall, these findings significantly advance the understanding of Chinese adolescents’ current identity status and the relationship between it and psychosocial adjustment. By identifying adolescents’ profiles of identity statuses and elucidating their associations with psychosocial adjustment, this research provides valuable insights that can inform the development of interventions and support efforts aimed at promoting subjective well-being and resilience, and mitigating anxiety among Chinese adolescents. This research highlights the necessity to enhance students’ education on self-identity encouraging them to contemplate their future aspirations, academic pursuits, and career choices.

## Figures and Tables

**Table 1:** Descriptive Data and Correlations for the Study Variables (*n* = 4666).

Study Variables	*M* ± *SD*	1	2	3	4	5	6	7	8	9
1. Score of U-MICS	3.82 ± 0.70	1								
2. Commitment	4.12 ± 0.82	0.87 ***	1							
3. In-depth exploration	3.81 ± 0.75	0.96 ***	0.78 ***	1						
4. Reconsideration of commitment	3.34 ± 0.94	0.73 ***	0.34 ***	0.66 ***	1					
5. Total resilience	3.55 ± 0.55	0.44 ***	0.61 ***	0.40 ***	0.02	1				
6. Personal strength	3.67 ± 0.71	0.45 ***	0.59 ***	0.41 ***	0.06 ***	0.95 ***	1			
7. Support strength	3.40 ± 0.49	0.31 ***	0.48 ***	0.27 ***	−0.06 **	0.82 ***	0.60 ***	1		
8. Anxiety	33.94 ± 9.32	−0.18 ***	−0.24 ***	−0.15 ***	−0.05 **	−0.34 ***	−0.35 ***	−0.24 ***	1	
9. Subjective well-being	5.25 ± 1.49	0.40 ***	0.51 ***	0.36 ***	0.09 ***	0.62 ***	0.61 ***	0.47 ***	−0.33 ***	1

** *p* < 0.01, *** *p* < 0.001.

**Table 2:** Fit Indices for the Latent Profile Analyses (*n* = 4666).

Profiles	LL	AIC	BIC	SSA-BIC	Entropy	LMR-LRT	BLRT	Percentage of Sample
2	−16,080.88	32,181.77	32,246.25	32,214.47	0.830	3116.13 ***	3208.35 ***	34.2/65.8
3	−13,741.25	27,510.49	27,600.77	27,556.28	1.000	4544.78	4679.28 ***	32.7/46.3/21.0
4	−8845.11	17,726.23	17,842.30	17,785.10	1.000	9510.81	9792.26 ***	30.5/2.2/46.3/20.9
5	−8589.86	17,223.71	17,365.57	17,295.66	0.970	502.03 *	501.52 ***	2.2/46.3/26.8/3.7/20.9
6	−8112.38	16,276.76	16,444.41	16,361.79	0.990	927.50	954.95 ***	2.2/17.1/17.7/13.4/28.6/20.9
7	−7686.99	15,433.97	15,627.41	15,532.09	1.000	839.75	850.79 ***	3.7/2.2/9.9/17.7/20.9/17.0/28.6

LL = model log likelihood; AIC = Akaike information criterion; BIC = Bayesian information criterion; SSA-BIC = sample-size adjusted BIC; LMR-LRT = Lo–Mendell–Rubin likelihood ratio test; BLRT = bootstrap likelihood ratio test; * *p* < 0.05, *** *p* < 0.001.

**Table 3:** Discriminant Analyses (*n* = 4666).

		Clusters of Discriminant Analyses					
		Cluster 1	Cluster 2	Cluster 3	Cluster 4	Cluster 5	Total
Clusters of LPA	Cluster 1	91 (89.2%)	0 (0%)	1 (1.0%)	10 (9.8%)	0 (0%)	102
	Cluster 2	0 (0%)	1954 (90.4%)	47 (2.2%)	75 (3.5%)	85 (3.9%)	2161
	Cluster 3	0 (0%)	0 (0%)	1171 (93.5%)	81 (6.5%)	0 (0%)	1252
	Cluster 4	0 (0%)	0 (0%)	0 (0%)	174 (100%)	0 (0%)	174
	Cluster 5	0 (0%)	0 (0%)	0 (0%)	0 (0%)	977 (100%)	977
	Total	91	1954	1219	340	1062	4666

**Table 4:** Associations of Gender and Age with Identity Statuses and Differences in Anxiety, Subjective Well-Being, and Resilience Measures (*n* = 4666).

Demographic Variables	Diffusion *n* = 102	Undifferentiated *n* = 2161	Avoidant *n* = 1252	Foreclosure *n* = 174	Searching Moratorium *n* = 977	*F*/χ^2^	η_p_^2^/Cramer’s *V*
Male, %	61.76	45.58	49.28	31.61	63.05	112.876 ***	0.156
Female, %	38.24	54.42	50.72	68.39	36.45		
Age, Mean (SD)	18.32 ± 0.56	18.37 ± 0.53	18.36 ± 0.53	18.35 ± 0.51	18.38 ± 0.56	0.491 (ns)	
Anxiety	34.60 ± 11.71 ^a^	34.21 ± 8.59 ^b^	35.94 ± 8.86 ^a^	31.11 ± 7.85 ^c^	31.19 ± 10.57 ^c^	36.846 ***	0.031
Subjective well-being	4.45 ± 1.95 ^c^	5.36 ± 1.36 ^b^	4.40 ± 1.32 ^c^	6.17 ± 1.21 ^a^	6.05 ± 1.37 ^a^	225.021 ***	0.162
Personal strength	3.18 ± 0.83 ^d^	3.71 ± 0.65 ^c^	3.22 ± 0.50 ^d^	4.30 ± 0.62 ^a^	4.10 ± 0.68 ^b^	323.753 ***	0.218
Support strength	3.05 ± 0.53 ^d^	3.44 ± 0.48 ^c^	3.16 ± 0.41 ^d^	3.78 ± 0.44 ^a^	3.57 ± 0.49 ^a^	166.801 ***	0.125
Total resilience	3.12 ± 0.63 ^d^	3.59 ± 0.51 ^c^	3.19 ± 0.39 ^d^	4.07 ± 0.47 ^a^	3.86 ± 0.53 ^b^	329.915 ***	0.221

ns = not significant. *** *p* < 0.001. Values are presented as mean ± standard deviation. A cluster mean is significantly different from another mean if they have different superscripts. A mean without a superscript is not significantly different from any other mean. a > b > c > d.

## Data Availability

The dataset used in the study is not publicly available but is available from the corresponding author on reasonable request, due to privacy or ethical restrictions.

## Abbreviations

The following abbreviations are used in this manuscript:

AIC	Akaike information criterion
ANOVA	analysis of variance
BIC	Bayesian information criterion
BLRT	bootstrap likelihood ratio test
df	degrees of freedom
IWB	Index of Well-Being
LL	log likelihood
LMR-LRT	Lo–Mendell–Rubin likelihood ratio test
LPA	Latent Profile Analysis
MANOVA	multivariate analysis of variance
M	mean
RSCA	Resilience Scale for Chinese Adolescents
SAS	Self-rating Anxiety Scale
SD	standard deviation
SSA-BIC	sample-size adjusted Bayesian information criterion
U-MICS	Utrecht-Management of Identity Commitments Scale
